# Alterations in Gut Vitamin and Amino Acid Metabolism are Associated with Symptoms and Neurodevelopment in Children with Autism Spectrum Disorder

**DOI:** 10.1007/s10803-021-05066-w

**Published:** 2021-07-14

**Authors:** Jiang Zhu, Xueying Hua, Ting Yang, Min Guo, Qiu Li, Lu Xiao, Ling Li, Jie Chen, Tingyu Li

**Affiliations:** 1 Children’s Nutrition Research Center, Children’s Hospital of Chongqing Medical University, Chongqing Key Laboratory of Childhood Nutrition and Health, Chongqing, 400014 China; 2 Ministry of Education Key Laboratory of Child Development and Disorders, China International Science and Technology Cooperation Base of Child Development and Critical Disorders, National Clinical Research Center for Child Health and Disorders, Chongqing, 400014 China; 3 Department of Neonatology, Chongqing Medical University Affiliated Children Hospital, Chongqing, 400014 China; 4 Department of Children Rehabilitation, Hainan Women and Children’s Medical Center, Hainan, 570100 China; 5 Director of Key Lab of Ministry of Education, Children’s Nutrition Research Center, Children’s Hospital of Chongqing Medical University, No.136 Zhongshan Er Road, Yuzhong District, Chongqing, 400014 China

**Keywords:** Autism, Metabolomics, Metabolism, Vitamin, Symptoms, Children

## Abstract

Metabolic disturbance may be implicated in the pathogenesis of autism. This study aimed to investigate the gut metabolomic profiles of autistic children and to analyze potential interaction between gut metabolites with autistic symptoms and neurodevelopment levels. We involved 120 autistic and 60 neurotypical children. Autistic symptoms and neurodevelopment levels were assessed. Fecal samples were analyzed using untargeted liquid chromatography-tandem mass spectrometry methods. Our results showed the metabolic disturbances of autistic children involved in multiple vitamin and amino acid metabolism pathways, with the strongest enrichment identified for tryptophan metabolism, retinol metabolism, cysteine-methionine metabolism, and vitamin digestion and absorption. Differential gut metabolites were correlated to autistic symptoms and neurodevelopment levels. Our findings improved the understanding of the perturbations of metabolome networks in autism.

**Supplementary Information:**

The online version contains supplementary material available at 10.1007/s10803-021-05066-w.

## Introduction

Autism spectrum disorder (ASD) is a complex neurodevelopmental disorder characterized by early-appearing social communication deficits and restricted or repetitive behaviors (Hyman et al., 2020). Besides the core deficits, ASD is often accompanied with other developmental or behavioral disorders, sleep problems, and gastrointestinal (GI) symptoms (Hyman et al., 2020). Currently, the precise etiology and mechanism of ASD remain unclear, thus hindering the development of available laboratory diagnostic and effective cure for the condition (Muhle et al., 2018).

Accumulated evidence has supported metabolic disturbance may be implicated in the pathogenesis of ASD. Metabolomics studies of urines, plasma, and fecal samples from ASD patients have shown disturbances of metabolism related to amino acids, oxidative stress, purine intermediates, and gut microbiota (Glinton & Elsea, 2019; Shen et al., 2020; Mohamadkhani, 2018; Kang et al., 2018). Despite the high interest, previous studies are mainly focused on the metabolomic analysis of urines and blood, and analysis of gut microbiota composition, while studies of fecal metabolism are relatively rare in the context of ASD (Kang et al., 2018; Shen et al., 2020).

Gut metabolomics can provide comprehensive information about the final products of interactions among dietary intake, metabolism, and microbial functions. Studies have shown that short-chain fatty acids (SCFAs) altered in the gut of ASD children (Liu et al., 2019; Thomas et al., 2012), and SCFAs could regulate gut immunity and genes expression of the host (Chang et al., 2014). Abnormal glutamate and γ-aminobutyric acid (GABA) metabolism were observed in the feces of children with ASD, which may influence excitation-inhibition balance (Kang et al., 2018; Wang et al., 2019). Abnormal tryptophan metabolism and increased serotonin (5-hydroxytryptamine, 5-HT) have been observed in in the gut of ASD patients (Muller et al., 2016). Also, isopropanol and phenol substances, including phenol and *p*-cresol, were found higher in fecal of children with ASD (De Angelis et al., 2013; Kang et al., 2018).

Although the above reports suggest that altered gut metabolomics may contribute to the pathogenesis of ASD, the specific alterations in individual compounds were inconsistent between studies owing to multiple potential confounders (e.g., ethnicity, age, diet, disease, medicine, and methodology used) that can influence the metabolism outcomes. Inconsistent and scattered changes in single metabolites have a limited role in elucidating the pathophysiology of ASD. Therefore, a comprehensive interpretation of the metabolism pathway network is required.

This study aimed to determine the gut metabolomic profiles of children with ASD and identify the potential associations of gut metabolites with ASD symptoms and neurodevelopment levels. We analyzed the fecal metabolomic profiles of preschool children with ASD and age-, sex-, region- matched typically developing (TD) children with liquid chromatography-tandem mass spectrometry (LC–MS/MS) methods. We found that the differential metabolites between the ASD and TD groups were mainly involved in multiple vitamin and amino acid metabolism pathways. We also investigated the possible correlations of the altered gut metabolites with symptoms and neurodevelopment levels of ASD children, and postulated the interconnection of vitamins and amino acids in the metabolism network of ASD.

## Subjects and Methods

### Subject Selection

A total of 120 children with ASD (ages, 2–6 years) were enrolled for this study from the Maternal and Child Care Health Hospital of Hainan Province, China, after a comprehensive assessment. The inclusion criteria were a diagnosis of ASD made by a developmental pediatrician through a series of structured interviews according to the Diagnostic and Statistical Manual of Mental Disorders (Fifth Edition, DSM-5) criteria (American Psychiatric Association, 2013). The Childhood Autism Rating Scale (CARS) (Rellini et al., 2004) was used to assist diagnosis (scores > 30). The exclusion criteria included other developmental disorders, neurological or psychiatric diseases, genetic metabolic disease, major physical illness, a recent history of (i.e., within 3 months before sampling) infection, special diets, or antibiotic/probiotic use.

ASD symptoms were assessed using the Autism Behavior Checklist (ABC) (Rellini et al., 2004), Social Responsiveness Scale (SRS) (Cen et al., 2017) and CARS, and higher scores indicated more severe symptoms. Neurodevelopment level in ASD children was assessed using the revised Gesell Developmental Scale (GDS) (Jin et al., 2007), which is extensively used in China to evaluate cognitive and behavioral development. Development quotient scores (DQ) of GDS were used to assess the levels of intellectual and behavioral development. DQ scores < 75 indicated developmental delay, and the lower the DQ score, the more severe the developmental delay. Gastrointestinal (GI) symptoms were evaluated with the six-item Gastrointestinal Severity Index (6-GSI), and higher scores indicated more severe GI symptoms.

A control group of 60 TD children was recruited and matched to the ASD group by age, gender, and region. The TD children underwent health examinations at the Department of Child Health in the Maternal and Child Care Health Hospital of Hainan Province. All control subjects were healthy, and showed no sign of developmental disorders, psychiatric diseases, or GI symptoms. Other exclusion criteria were the same as those for the ASD group.

Participation in this research was voluntary. The study protocol was approved by the Medical Ethic Committee. This cross-sectional study was based on a clinical trial which was registered in the Chinese Clinical Trial Registry (ChiCTR-ROC-14005442).

### Fecal Sample Collection and LC–MS Metabolomics Analysis

#### Fecal Sample Collection and Metabolites Extraction

Fresh fecal samples from each participant were placed in sterile tubes, immediately frozen, and stored at − 80 °C until metabolomics analysis. The 100 mg of stool for each sample was separately ground with liquid nitrogen, and the resulting homogenate was resuspended in prechilled 80% methanol and 0.1% formic acid by vortexing thoroughly. The samples were incubated on ice for 5 min and then centrifuged at 15,000 rpm/min at 4 °C for 5 min. The supernatant was diluted with LC–MS-grade water (final concentration, 60% methanol), transferred into a fresh Eppendorf tube through a 0.22-μm filter, and centrifuged again at 15,000 rpm/min at 4 °C for 10 min. Finally, the filtrate was injected into the LC–MS/MS system for analysis.

#### LC–MS/MS Analysis

LC–MS/MS analyses were performed using a Vanquish UHPLC system (Thermo Fisher, USA) and an Orbitrap Q Exactive HF-X mass spectrometer (Thermo Fisher). Briefly, the metabolites were separated and characterized using LC system and further detected with MS system. Samples were injected onto an Hyperil Gold column (100 × 2.1 mm, 1.9 μm) at a flow rate of 0.2 mL/min and separated using a 16-min linear gradient. The eluents for the positive polarity mode were 0.1% formic acid in water (eluent A) and methanol (eluent B). The eluents for the negative polarity mode were 5 mM ammonium acetate, pH 9.0 (eluent A) and methanol (eluent B). The solvent gradient was set as follows: 2% B,1.5 min; 2 – 100% B, 12.0 min; 100% B, 14.0 min; 100 – 2% B, 14.1 min; and 2% B, 16 min. The Q Exactive HF-X mass spectrometer was operated in the positive/negative polarity mode with 3.2 kV spray voltage, 35 arb sheath gas-flow rate, 10 arb aux gas-flow rate, and 320 °C capillary temperature.

#### Metabolite Analysis

Compound Discoverer *v*3.0 (CD 3.0, Thermo Fisher) was used to process and normalize the raw data files generated by UHPLC–MS/MS and to perform peak alignment, peak selection, and quantification for each metabolite. The main parameters were set as follows: retention time tolerance, 0.2 min; actual mass tolerance, 5 ppm; signal intensity tolerance, 30%; signal/noise ratio, 3; and minimum intensity, 100,000. Peak intensities were normalized against the total spectral intensity, and normalized data were used to predict the molecular formula based on additive ions, molecular ion peaks, and fragment ions. Peaks were matched with the mzCloud (https://www.mzcloud.org/) and ChemSpider (http://www.chemspider.com/) databases to obtain accurate qualitative and relative quantitative results.

The normalized metabolism data were analyzed using the CentOS (release 6.6), R (*v*R-3.4.3), and SPSS (*v*19.0, USA). With individual metabolites dataset, Partial least squares discriminant analysis (PLS-DA) models were built to visualize the metabolic alteration patterns between the ASD and TD groups. Furthermore, the cross-validation analysis of variance was performed to assess the reliability of the models. Differential metabolites between the two groups were selected using combined multivariate and univariate analyses. Gut metabolites with a fold change > 1.5, a variable importance in projection score (VIP score) > 1, and a false discovery rate-corrected *p* value < 0.05 for the Student’s *t* test or Mann–Whitney *U* test were considered significantly differentially expressed between the two groups. To further demonstrate the biological functions of the differential metabolites, the Kyoto Encyclopedia of Genes and Genomes (KEGG) pathways enrichment analysis was performed (http://www.genome.jp/kegg/). A hypergeometric test was used to assess the significance of the enriched KEGG pathway.

Metabolomics analysis was carried out according to the standard protocols recommended by Novogene Technology Co., Ltd. (Beijing, China).

### Statistical Analysis

Demographics and clinical assessment data were analyzed using SPSS (*v*19.0). Continuous variables were described as the means with standard deviations or medians (interquartile ranges) as appropriate, and categorical variables were described as percentages. The two-tailed Student’s *t *test, Mann–Whitney *U* test, and the chi-square test were used to compare between groups. Correlations of metabolites levels with clinical assessment scores were analyzed by Spearman correlation. *p* value < 0.05 indicated statistical significance.

## Results

### Subject Characteristics

A total of 120 ASD children (ages, 2–6 years) and 60 TD children were enrolled for this study. The demographic and clinical features of the participants are presented in Table 1. There were no significant differences in age-gender composition and z-score of the body mass index (BMI) between the two groups. Of the 120 children with ASD, 80 (66.67%) showed food selectivity, and 58 (48.33%) had GI symptoms.

**Table 1:** Demographic and clinical characteristics of participants

	TD	ASD	*p* value
Age (years), mean ± SD	4.01 ± 1.12	3.86 ± 1.03	0.2182
Sex (male/female)	39/21	99/21	0.079
Family annual income per capita (RMB), n (%)			
≤20,000	31 (51.67)	67 (55.83)	0.597
> 20,000	29 (48.33)	53 (44.17)	
Height (ZHA)	0.01 ± 0.94	−0.14 ± 1.0	0.2429
Weight (ZWA)	−0.02 ± 0.98	0.06 ± 0.96	0.5771
BMI (ZBMI)	0.26 ± 0.92	0.33 ± 1.07	0.6646
Picky eating, n (%)	26 (43.33)	80 (66.67)	0.003^**^
GI symptoms, n (%)	0	58(48.33)	
ABC			
Sensory	–	8.37 ± 5.04	
Social withdrawal	–	14.13 ± 7.74	
Stereotypic behavior	–	7.63 ± 7.01	
Inappropriate speech	–	13.57 ± 5.98	
Laggard daily living ability	–	11.28 ± 5.18	
Total ABC scores	–	54.98 ± 22.74	
SRS			
Social awareness	–	11.83 ± 3.09	
Social cognition	–	18.68 ± 4.52	
Social communication	–	33.29 ± 8.82	
Social motivation	–	14.96 ± 4.4	
Autistic mannerisms	–	13.71 ± 5.58	
Total SRS scores	–	92.47 ± 21.65	
CARS	–	37.18 ± 5.86	
GDS			
Adaptive behavior	–	57.01 ± 17.94	
Gross motor	–	64 ± 14.22	
Fine motor	–	57.45 ± 17.05	
Language	–	43.62 ± 19.97	
Personal-social behavior	–	48.47 ± 15.24	

The two-tailed Student’s *t* test, and the chi-square test were used for analysis

*TD* typically developing, *ASD* autism spectrum disorders.

^**^p < 0.01

### Alterations in Gut Metabolism Profiles of ASD Children

**Fig. 1:**
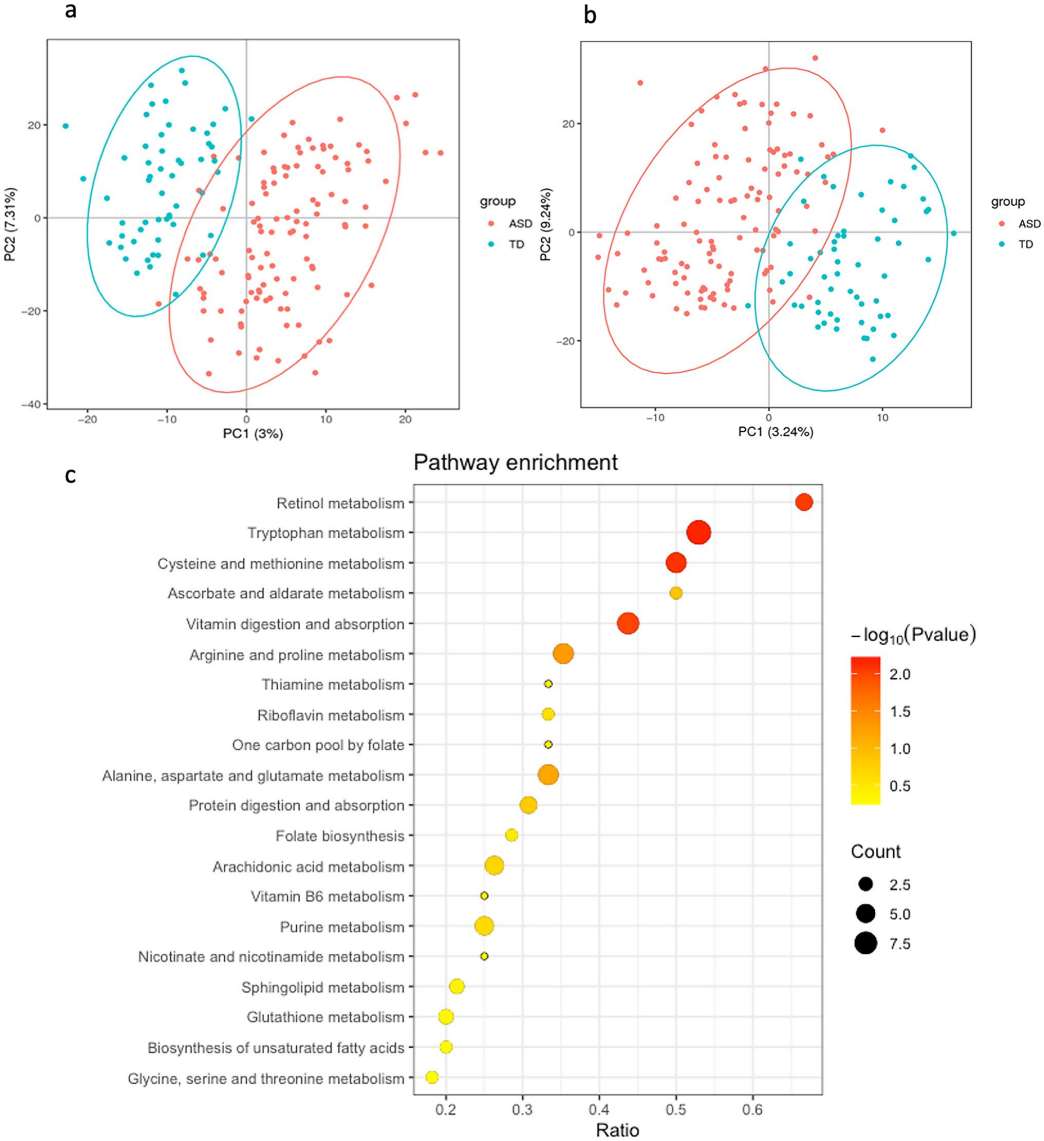
Alterations of the gut metabolome in children with ASD compared with TD children. **a**, **b** The clustering analyses of partial least-squares discriminant analysis (PLS-DA) of gut metabolome data in the positive ion mode (**a**) and negative ion mode (**b**). **c** Top 20 KEGG pathways enriched by differential gut metabolites between the ASD and TD children. Count, the number of differential metabolites in the pathway. Ratio, the ratio of number of differential metabolites to all detected metabolites in the pathway. *p* value, *p* value of hypergeometric test. *ASD* autism spectrum disorders, *TD* typically developing

To explore the gut metabolic patterns associated with ASD status, a fecal metabolome analysis was performed by LC–MS/MS method. A total of 6936 peaks of compounds were obtained, among which 4531 were explored in the positive ion mode (ESI+) and 2405 in the negative ion mode (ESI−). PLS-DA showed that the ASD and TD groups were well clustered with particular metabolic profiles for each (ESI+ : R^2^Y = 0.73, Q^2^ = 0.61, *p* < 0.0001; ESI−: R^2^Y = 0.76, Q^2^ = 0.65, *p* < 0.0001; Fig. 1a, b). The permutation test with *p* < 0.001 indicated that the classification of global metabolite profiles between the ASD and TD groups was significantly different. We identified 96 differential metabolites between the ASD and TD groups, including 35 significantly increased metabolites and 61 significantly decreased metabolites in the ASD group (Table S1).

KEGG pathway analysis revealed 27 KEGG pathways associated with ASD status (Table S2). Interestingly, the differential metabolites were mainly enriched in multiple vitamin and amino acid metabolism pathways, with the strongest enrichment identified for tryptophan metabolism (*p* = 0.0006), retinol metabolism (*p* = 0.009), cysteine and methionine metabolism (*p* = 0.008), and vitamin digestion and absorption (*p* = 0.01; Fig. 1c). In addition, some differential metabolites were involved in arachidonic acid, steroid hormone, citrate cycle, and purine metabolism.

**Fig. 2:**
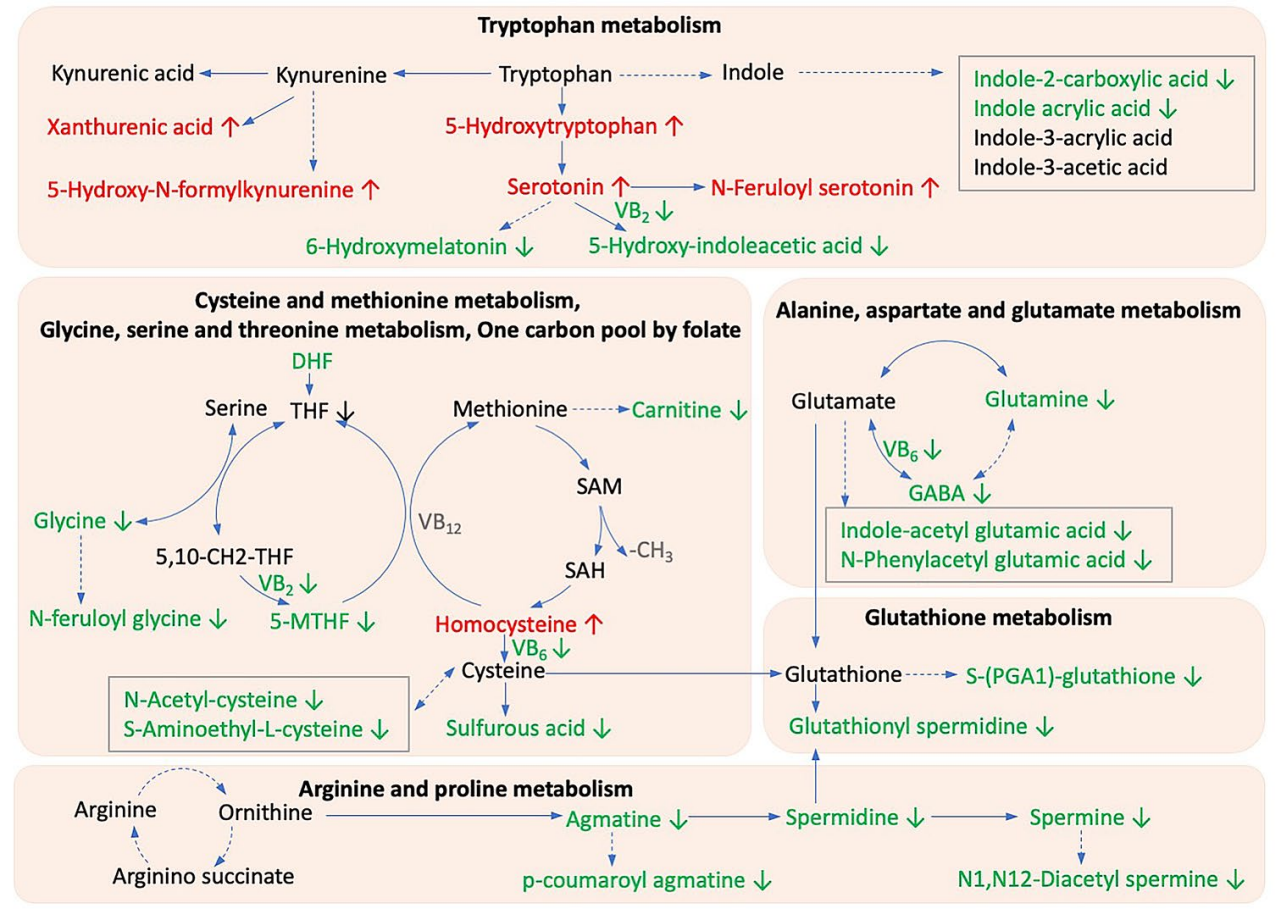
Metabolism pathway networks of the differential metabolites between the ASD and TD group. Gut metabolites with a fold change > 1.5, a variable importance in projection score(VIP) > 1, and a FDR-corrected *p* values < 0.05 for the Student’s *t* test or Mann–Whitney *U* test were considered significantly differentially expressed between the two groups. Red font (↑), metabolites increased in the ASD group; Green font (↓), metabolites decreased in the ASD group; black font, no significant difference between the ASD and TD groups; grey font, undetected. DHF, dihydrofolate; 5-MTHF, 5-methyltrahydrofolate; SAM, S-adenosylmethionine; SAH, S-adenosylhomocysteine. *ASD* autism spectrum disorders; *TD* typically developing

Disturbances in various vitamin metabolism pathways were found in children with ASD, as shown in Table 2 and Fig. 2. In the retinol metabolism pathway, abnormal levels of precursors and intermediates of vitamin A were found in the ASD group. The 4′-apo-beta-carotenal, b,e-carotene-3,3′-diol, and retinal levels were increased, while retinol level was decreased. The concentrations of multiple B vitamins and their derivatives were decreased, including thiamine pyrophosphate (TPP), riboflavin (vitamin B2) and its intermediate lumichrome, phosphopantothenic acid (vitamin B5 derivative), pyridoxamine (vitamin B6), 1,4,5,6-tetrahydro-6-oxonicotinic acid, dihydrofolate (DHF), and 5-methyltetrahydrofolate(5-MTHF). The vitamin C level was also decreased in the children with ASD.

**Table 2:** Aberrant gut metabolites relevant to vitamins and cofactors in children with ASD

Vitamins metabolites	Metabolism pathway	Fold change^a^	*p* value	Regulation mode^b^
4′-Apo-beta-carotenal	Vitamin digestion and absorption, retinol metabolism	1.55	9.24E-05	up
b,e-Carotene-3,3′-diol	Vitamin digestion and absorption, retinol metabolism	1.56	0.0043	up
All-trans-retinal	Retinol metabolism	1.56	0.0262	up
Retinol	Retinol metabolism	0.64	0.0149	down
Tocopherol	Vitamin E metabolism	3.19	0.002	up
Thiamine pyrophosphate	Thiamine metabolism	0.45	0.0224	down
Riboflavin tetrabutyrate	Riboflavin metabolism	0.21	0.0085	down
(+)-Riboflavin	Riboflavin metabolism	0.62	0.0305	down
Lumichrome	Riboflavin metabolism	0.64	0.005	down
Pyridoxamine	Vitamin digestion and absorption, vitamin B6 metabolism	0.63	0.0035	down
Phosphopantothenic acid	Vitamin B5 metabolism	0.64	0.0181	down
5-Methyltetrahydrofolate	Folate biosynthesis	0.56	0.0068	down
Dihydrofolic acid	Vitamin digestion and absorption, folate biosynthesis	0.49	0.006	down
1,4,5,6-Tetrahydro-6-oxonicotinic acid	Nicotinate and nicotinamide metabolism	0.66	0.0036	down
Vitamin C	Vitamin digestion and absorption, ascorbate and aldarate metabolism	0.58	0.0148	down
L-Ascorbic acid	Ascorbate and aldarate metabolism	0.51	0.0031	down

^**ab**^ Fold change and regulation mode in the ASD group compared with the typically developing group

*ASD* autism spectrum disorders

Aberrant amino acid metabolisms were also associated with ASD (Fig. 2), and tryptophan metabolism pathway was the most affected. The concentrations of xanthurenic acid, 5-hydroxy-*N*-formylkynurenine, 5-hydroxytryptophan (5-HTP), serotonin (5-hydroxytryptamine, 5-HT), and *N*-feruloyl serotonin were significantly increased in the ASD group, while the 6-hydroxymelatonin and 5-hydroxyindoleacetic acid (5-HIAA) levels were decreased in the ASD group. Besides, some indole derivatives, including indole acrylic acid and indole-2-carboxylic acid, were decreased in the ASD group. The cysteine-methionine metabolism pathway is closely related to folate metabolism. Both pathways showed abnormalities in the ASD group, with lower levels of DHF, 5-MTHF, *N*-acetylcysteine (NAC), and *S*-aminoethyl-*L*-cysteine, and excessive accumulation of homocysteine (Hcy). In the case of arginine metabolism pathway, the concentrations of polyamines, including agmatine, spermine, and glutathione spermidine, were lower in children with ASD. We also found abnormal glutamate and glycine metabolism, with decreased glutamine, GABA, and glycine levels in children with ASD.

Disturbances were also detected in the biologically active metabolites of arachidonic acid, which are crucial regulators of oxidative stress and inflammation. Arachidic acid and 20-hydroxy-leukotriene E4 levels were increased, while leukotriene B4 and 5-trans prostaglandin F2β levels were decreased in children with ASD. Besides, the purine metabolite 8-hydroxy-deoxyguanosine (8-OHdG), which is a sensitive marker of oxidative DNA damage (Valavanidis et al., 2009), was significantly increased (6.86-fold; *p* = 0.001) in the ASD group.

### Correlation of Gut Metabolites with ASD Symptoms and Neurodevelopment Levels

**Fig. 3:**
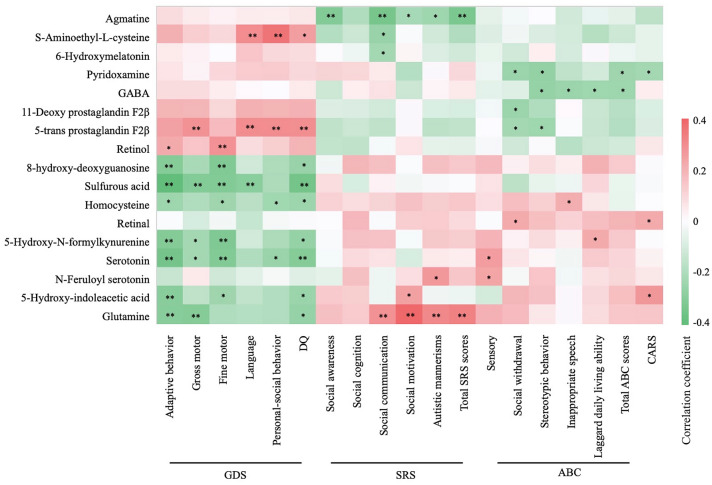
Correlations of gut metabolites with ASD symptoms and neurodevelopment levels. The Spearman correlation coefficient is indicated by a color gradient from green (negative correlation) to red (positive correlation). The * symbol in each lattice represent a significant correlation. *p < 0.05, **p < 0.01. *GDS* Gesell Developmental Scale, *ABC* autism behavior checklist, *SRS* Social Responsiveness Scale, *CARS* Childhood Autism Rating Scale, *DQ* development quotient scores.

Spearman correlation analysis was performed to explore the potential links between key fecal metabolites and clinical assessment scores of children with ASD. Agmatine, *S*-aminoethyl-*L*-cysteine, 6-Hydroxymelatonin, pyridoxamine, GABA, and 5-trans prostaglandin F2β were negatively correlated with partial subscales or total ABC, SRS or CARS scores. Conversely, retinal, Hcy, serotonin, N-feruloyl serotonin, and 5-HIAA in the gut were positively correlated with ASD symptoms. *S*-aminoethyl-*L*-cysteine, 5-trans prostaglandin F2β, and retinol were positively correlated with neurodevelopment scores, while 8-OHdG, Hcy, 5-hydroxy-*N*-formylkynurenine, and serotonin were negatively correlated with neurodevelopment scores (Fig. 3; Table S3).

### Impact of GI Problems on the Metabolic Patterns in ASD Children

Gastrointestinal (GI) problems is often co-occurred with ASD and we found 48.33% of ASD children suffered from GI symptoms. The impact of GI problems on the gut metabolic patterns of children with ASD was evaluated. The supervised PLS-DA showed that the ASD children with GI symptoms (ASD-GI, n = 58) and non-GI symptoms (ASD-nonGI, n = 62) were not partly clustered with specific metabolic profiles for each (ESI+ : R^2^Y = 0.27, Q^2^ = 0.10, *p* = 0.14; ESI-: R^2^Y = 0.25, Q^2^ = 0.06, *p* = 0.045),while the metabolic profiles of the both groups were different from the TD group (all *p* value < 0.001; Figure S1a-b). We found most differential metabolites revealed between ASD and TD groups were not different between ASD-GI and ASD-nonGI groups (data not shown). The results showed GI problems had a limited impact on the metabolic patterns and presence of differential metabolites.

Spearman correlation analysis revealed that serotonin and N-feruloyl serotonin levels were positively correlated with 6-GSI scores (rs = 0.185, *p* = 0.044; rs = 0.276, *p* = 0.002). We found a positive association between dihydrofolic acid levels and 6-GSI scores (rs = 0.341, *p* < 0.001), which indicated GI problems may affect the absorption of folic acid.

## Discussion

This study showed that gut metabolomic profiles significantly differed between young children with ASD and TD children. The differential fecal metabolites were mainly involved in vitamin and amino acid metabolism pathways, with the strongest enrichment identified for tryptophan metabolism, retinol metabolism, cysteine and methionine metabolism, and vitamin digestion and absorption. Some metabolic perturbations were associated with ASD symptoms and neurodevelopment levels, and may contribute to the pathogenesis of ASD through the gut-brain axis.

Vitamin A is required for functional systemic development in children (McLean et al., 2020), and studies have showed that children with ASD are more vulnerable than neurotypical children to vitamin A deficiency (Guo et al., 2018; Ranjan et al. 2015). In our study, the increased 4′-apo-beta-carotenal and b,e-carotene-3,3′-diol levels and decreased retinol level may indicate that children with ASD had a decreased capacity for the absorption and bioconversion of plant-origin precursors of vitamin A. Vitamin A has three active forms in humans: retinal, retinol, and retinoic acid (RA) (Kedishvili, 2016). RA, the main active form of vitamin A, is a crucial signaling molecule that regulates multiple fundamental biological processes (Kedishvili, 2016). The increased retinal level in our study may imply that the conversion of retinal to RA was suppressed in the gut of children with ASD. Excessive retinal may damage the nervous system. We found that in children with ASD, the retinol level was positively correlated with neurodevelopment levels, and the retinal level was positively correlated with the social withdrawal subscale of SRS. The ALDH1A family consists of key enzymes that oxidize retinal into RA, and XX Xu et al. (Xu et al., 2018) found that ASD patients with excessive UBE3A (an autism-related gene and molecule) may have congenital errors of retinol metabolism, as excessive UBE3A can inhibit ALDH1A activity and compromise the oxidation of retinal to RA. Moreover, the gut microbiota can participate in the alternative biotransformation of retinal to retinol or RA (Hong et al., 2016).

B vitamins are important cofactors implicated in multiple biochemical reactions. TPP, a derivative of thiamine (vitamin B1), is a cofactor of various enzymes in the mitochondria. Anwar A et al. (Anwar et al., 2016) found that plasma TPP concentrations were significantly lower in children with ASD than in controls. Consistent with this, we found lower levels of TPP in the feces of children with ASD than in TD children. Decreased TPP can lead to reduced mitochondrial anti-oxidative potential and energy production, and subsequently cellular damage (Altuner et al., 2013; Cinici et al., 2018). Vitamins B2 and B6 also participate in multiple amino acids metabolism processes. We found the level of pyridoxamine, a form of vitamin B6, was slightly negatively correlated to ABC and CARS scores.

The pathways of cysteine and methionine cycle, folate(vitamin B9) metabolism, and Hcy transsulfuration are interrelated and together constitute the folate-related metabolism pathway (Zou et al., 2019), which is critical for cell proliferation, DNA synthesis, immune function, and neural development (Sun et al., 2016). Vitamins B6 and B12 are cofactors in these biological processes. Decreased folate and vitamin B6 levels may lead to Hcy accumulation and decreased methyl production. Much of evidence suggested that folate deficit and excessive Hcy are risk factors for neural tube defects and neurodevelopmental disorders (Türksoy et al., 2014), and children with ASD have decreased folate levels and elevated Hcy levels in the blood and urine (Paşca et al., 2006; Yektaş et al., 2019). In our study, Hcy levels were negatively correlated with neurodevelopment scores, indicating the adverse impact of excessive Hcy on brain development and function. Moreover, NAC is an antioxidant with potential benefits in treating the irritability in children with ASD (Nikoo et al., 2015).

Abnormal tryptophan metabolism pathway in ASD has been reported in multiple studies, which was characterized by decreased tryptophan concentrations (Ormstad et al., 2018) and increased serotonin levels in the blood (Muller et al., 2016). In the gut, there are three main tryptophan metabolism pathways, which lead to kynurenine, serotonin, and indole derivatives (Agus et al., 2018; Kałużna-Czaplińska et al., 2019). Through the kynurenine pathway, kynurenic acid, xanthurenic acid, and quinolinic acid are generated (Agus et al., 2018). In our study, xanthurenic acid and 5-hydroxy-*N*-formylkynurenine levels were significantly increased in the ASD group. Vitamin B6 is a cofactor of kynureninase and kynurenine aminotransferase; therefore, the decrease of B6 may have contributed to the increased xanthurenic acid and 5-hydroxy-*N*-formylkynurenine levels. In the serotonin pathway, 5-HTP, serotonin, and *N*-feruloyl serotonin were significantly increased in feces of children with ASD, while 6-hydroxymelatonin and 5-HIAA were decreased. Reproducible evidence suggested serotonin-melatonin pathway in ASD is impaired, leading to hyperserotonemia and melatonin deficit in plasma (Abdulamir et al., 2018; Pang et al., 2014; Muller et al., 2016). However, few studies have reported altered tryptophan metabolism and serotonin-melatonin levels in the gut of ASD patients. Angelis et al. (De Angelis et al., 2013) found increased tryptophan and 3-methylindole levels in the feces of children with ASD. Dan Z et al. (Dan et al., 2020) also reported abnormal tryptophan metabolism in the gut of children with ASD. An mice model of autism found decreased serotonin in intestine mucosal (Golubeva et al., 2017). However, given that 95% of the serotonin in the body is generated in the intestine (Colle et al., 2020), it is likely that blood serotonin levels are correlated with enteric serotonin. Likewise, the GI tract, in addition to the pineal gland, is an important source of melatonin besides the pineal gland (Gagnon & Godbout, 2018). Melatonin can regulate sleep patterns, immunity, as well as GI function (Gagnon & Godbout, 2018). Serotonin can be catabolized to 5-HIAA, and this process depends on riboflavin (vitamin B2) as a cofactor, so riboflavin deficiency may be related to the increase of serotonin. Moreover, dysbiosis of the gut microbiota has been linked to abnormal tryptophan metabolism (Agus et al., 2018). We found a negative correlation between gut serotonin levels and neurodevelopment scores of ASD children, while serotonin and *N*-feruloyl serotonin levels were positively correlated with the sensory subscales of ABC and GI problems. Many studies have indicated that the blood serotonin levels are correlated with the severity of autism severity (Abdulamir et al., 2018). A balanced amount of enteric serotonin is beneficial to the functioning of the intestine, nervous system, and gut-brain axis, while excess serotonin may play a harmful role in the ASD progression.

We found decreased GABA, glutamine, glycine, and polyamines in fecal of children with ASD. GABA was negatively correlated with ABC scores, and agmatine was negatively correlated with SRS scores. These amino derivatives are crucial neurotransmitters or neuromodulators in the nervous system, and are important for immunity and inflammation (Ueland et al., 2017). GABA and glycine are inhibitory neurotransmitters, and their decrease may impact the excitation-inhibition balance of the nervous system (Nelson & Valakh, 2015). Our findings are partially supported by Kang DW et al. 2018 and Angelis et al. (De Angelis et al., 2013), who reported possibly lower GABA concentrations in the gut of children with ASD compared with healthy controls. Ford et al. (2020) found that aberrant glutamate and GABA processes were linked with impaired psychosocial function. Particularly, the synthesis of both GABA and glycine depend on vitamin B6 as a cofactor (Sato, 2018).

Biologically active metabolites of arachidonic acid showed disturbance, which are key regulators in oxidative stress and inflammation (Sergeant et al., 2016). Besides, 8-OHdG, a purine metabolite, is a sensitive marker of oxidative DNA damage (Valavanidis et al., 2009). Elevated 8-OHdG levels has been found in the cerebellar (Sajdel-Sulkowska et al., 2009) and urinary excretion (Ming et al., 2005) of ASD patients. In the present study, 8-OHdG was significantly increased (6.86-fold) in autistic children compared to TD children. These results indicates that children with ASD may have a higher risk of gastrointestinal damage by oxidative stress and inflammation.

Gut metabolism is the result of interactions of multiple genetic and environmental factors, including disease, microbiome, and diet (Alexander & Turnbaugh, 2020). Gut microbiota are important for gut metabolism, as microflora can produce vitamins and participate in the metabolism of numerous substances (Hong et al., 2016; LeBlanc et al., 2013). Picky eating is almost one of important characterizations of children with ASD, so inadequate intake from food could also partly explain the decreased in multiple vitamins and amino acids. In addition, GI problems may affect the absorption of nutrients. Furthermore, vitamin abnormalities/deficiencies may contribute to altered amino acid metabolism, for vitamins B are implicated in multiple biochemical reactions (Sato, 2018). Metabolic interventions for ASD include supplementation of prebiotics and probiotics, vitamins (e.g., A, C, D, B6, B12, folate), amino acids, and their derivatives (e.g., glycine, NAC) (Bjørklund et al. 2019; DeFilippis, 2018; Höfer et al., 2017; Mierau & Neumeyer, 2019). These approaches could sometimes correct intestinal dysbiosis and nutritional deficiencies in ASD, and partly improve the downstream metabolic consequences. However, these interventions were not always effective (Bjørklund et al., 2019), for some inborn errors of metabolism are hard to rectify, and single-compound supplementation may be insufficient to overcome the extensive abnormalities of metabolic networks in ASD. Therefore, detailed evaluation and individualized interventions for ASD children are required.

## Limitations

There are limitations in the present study. First, this cross-sectional study revealed correlations, but our data do not allow to prove the causation of symptoms and gut metabolites outcome. In addition, the correlations were not very strong (correlation coefficients, 0.2–0.4), as the metabolic disturbance is only one of many factors related to neurological function and ASD symptoms. Second, fecal metabolism may reflect the final results of the interactions of diet, microbiota, and intestinal function; however, the metabolic activity of each intestinal segment and the absorption and utilization of metabolites remain unclear, and it was difficult to distinguish whether the metabolites are derived from the host or the gut microbiota. Thus, the simultaneous analysis of fecal, intestinal contents, blood, microbiota, and other biological samples may lead to a deeper understanding of metabolomics networks. Third, our participants were preschool children from an island of China with a comparably biological backgrounds; therefore, these findings may not be generalizable to all ASD patients in different regions, races, and ages. Finally, ASD is a group of complex neurodevelopmental disorders, and studies involving different ASD subtypes and other related diseases are needed to evaluate the disease specificity of the metabolomic disturbances (age, sex, with our without food selectivity, developmental delay, etc.). Some metabolic disturbances may be nonspecific for various neurodevelopmental diseases and have an extensive impact on brain function and neurodevelopment.

## Conclusions

**Fig. 4:**
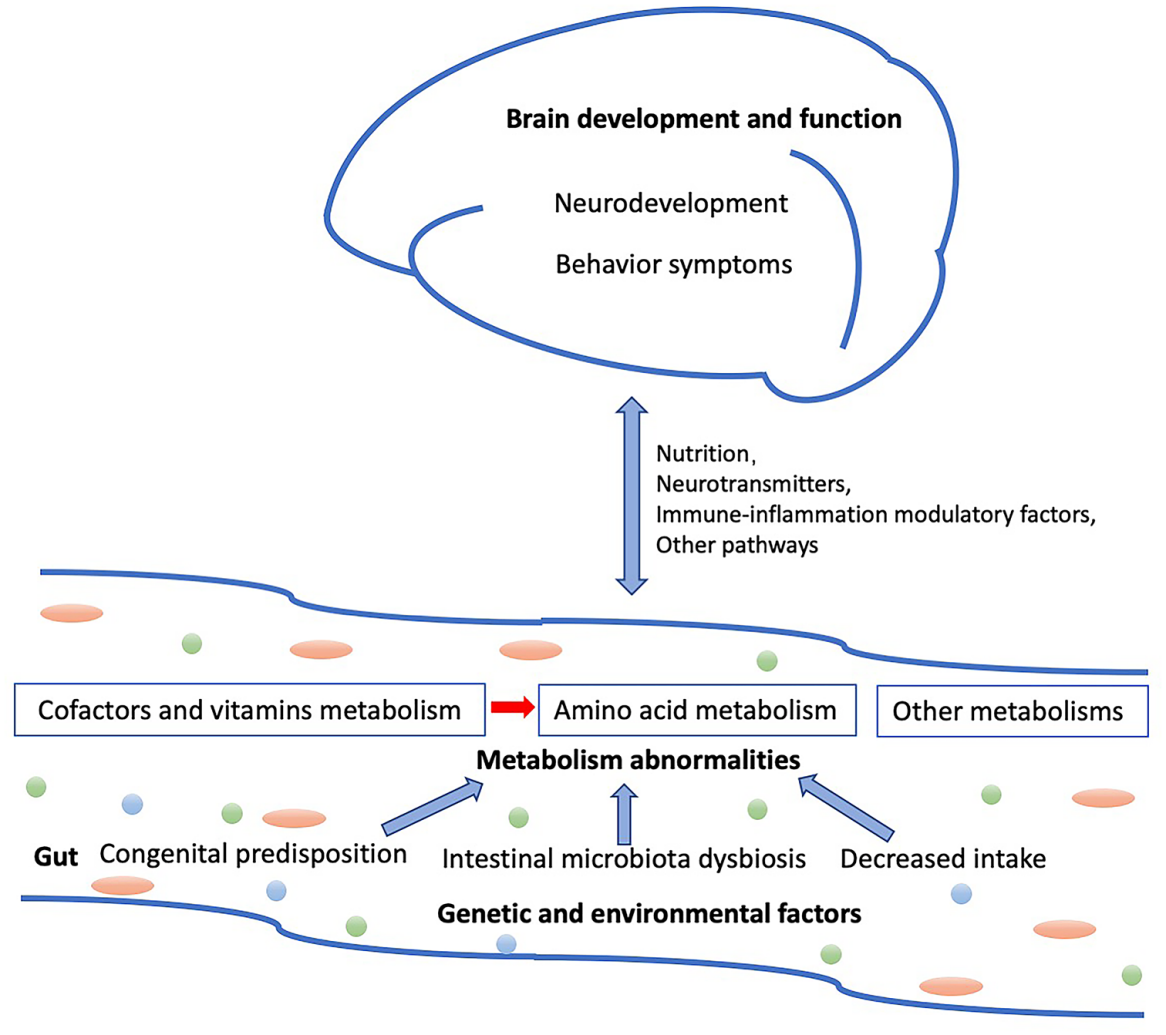
Hypothesis of interplay between the gut metabolism and the gut-brain axis in ASD. Aberrant of gut metabolism profiles in ASD may be the result of the interactions of multiple factors, including congenital errors in metabolism, decreased intake due to abnormal eating patterns, and intestinal microflora imbalance. In the interrelated metabolism networks, vitamin metabolism abnormalities and decreased vitamin intake may disturb the amino acid metabolism, as vitamins B are essential cofactors implicated in multiple biochemical reactions. The altered metabolites may affect the brain development and function, and subsequently behavior by nutrition, neurotransmitters, immune-inflammation modulatory, and other pathways

Children with ASD exhibit gut-metabolism perturbations that mainly involve amino acid and vitamin metabolism. These perturbations are related to ASD symptoms and neurodevelopment levels, and may be the result of the interaction of multiple factors, including congenital metabolism errors, decreased intake due to abnormal eating patterns, and intestinal microflora imbalance (Fig. 4). Notably, in the interrelated metabolism networks, vitamin metabolism abnormalities and decreased vitamin intake may disturb the amino acid metabolism, as B vitamins are essential cofactors implicated in multiple biochemical reactions. The differential metabolites may affect the brain development and function, and subsequently behavior via nutrition, neurotransmitter, immune-inflammation modulatory, and other pathways. Approaches such as nutritional supplementation and regulation of the intestinal flora may partially benefit the gut metabolism, nutritional status, and symptoms in ASD. It is essential to perform a detailed evaluation and provide comprehensive and individualized interventions for children with ASD. Our findings provided an extensive understanding of the disturbances in metabolism networks in ASD.

## Supplementary Information

Below is the link to the electronic supplementary material.

Supplementary file1 (PNG 201 kb) Gut metabolome profiles among ASD children with or without GI symptoms and TD children.

Supplementary file2 (XLSX 26 kb) List of identified differential gut metabolites between the ASD and TD children.

Supplementary file3 (XLSX 12 kb) KEGG pathway analysis of differential gut metabolites between the autistic and typical developing children.

Supplementary file4 (XLSX 24 kb) Correlations of gut metabolites with ASD symptoms and neurodevelopment levels.

## Data Availability

All data generated and analyzed in the current study are available from the corresponding author on reasonable request.

## Abbreviations

5-HIAA: 5-Hydroxyindoleacetic acid
5-HT: 5-Hydroxytryptamine
5-HTP: 5-Hydroxytryptophan
5-MTHF: 5-Methyltetrahydrofolate
8-OHdG: 8-Hydroxy-deoxyguanosine
ABC: Autism Behavior Checklist
ASD: Autism spectrum disorder
BMI: Body mass index
CARS: Childhood Autism Rating Scale
DHF: Dihydrofolate
DQ: Developmental quotient
GABA: γ-Aminobutyric acid
GDS: Gesell Developmental Scale
GI: Gastrointestinal
Hcy: Homocysteine
LC–MS: Liquid chromatography-mass spectrometry
NAC: N-acetylcysteine
PLS-DA: Partial least squares discriminant analysis
SRS: Social Responsiveness Scale
TD: Typically developing
TPP: Thiamine-pyrophosphate
VAD: Vitamin A deficiency
VIP: Variable important in projection
