# Supplementary figures and images for: Alterations in Gut Vitamin and Amino Acid Metabolism are Associated with Symptoms and Neurodevelopment in Children with Autism Spectrum Disorder
Source: J Autism Dev Disord. 2021 Jul 14;52(7):3116–28. doi: 10.1007/s10803-021-05066-w (PMC9213278; doi:10.1007/s10803-021-05066-w)

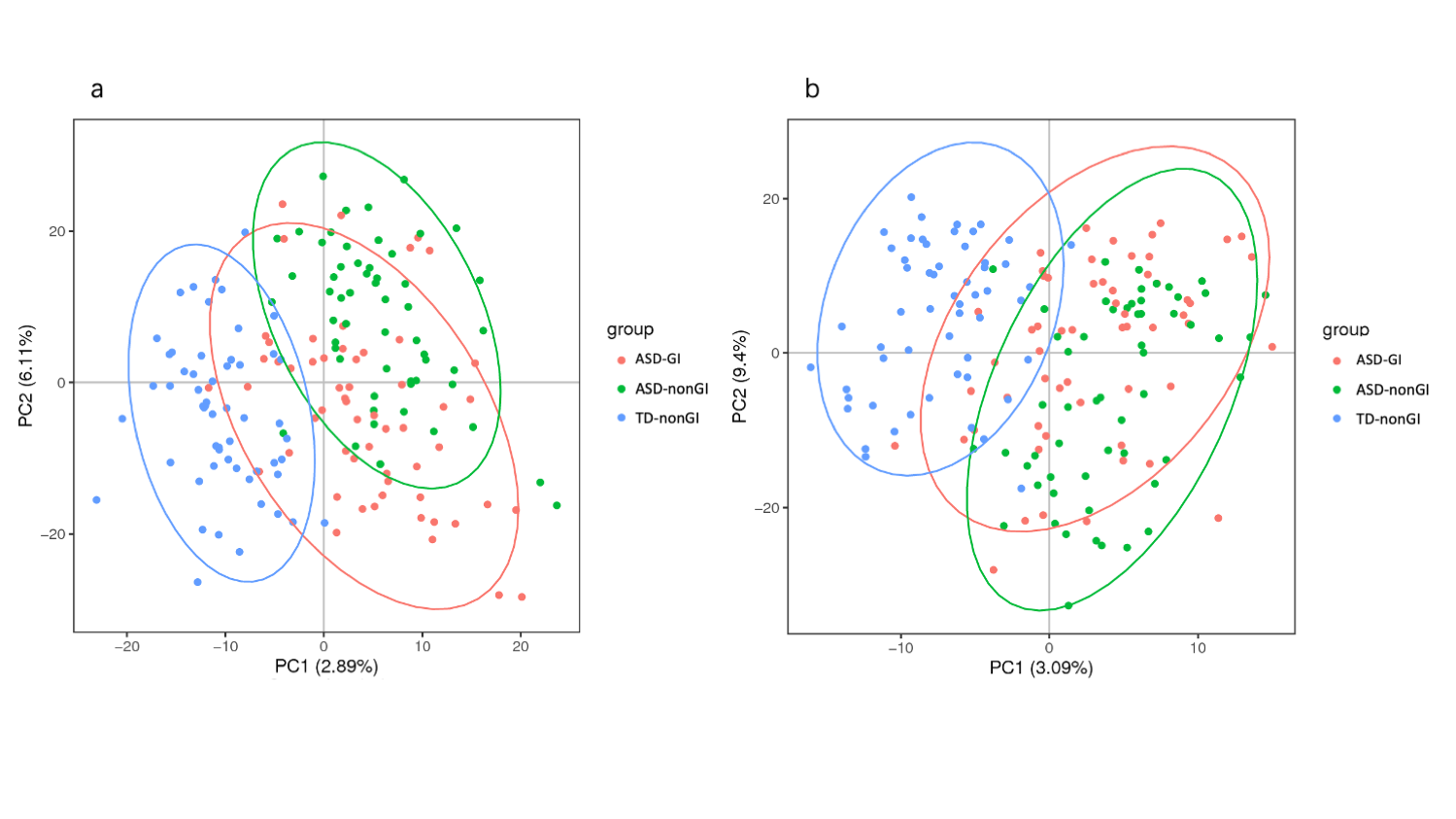

Supplement: Supplementary file 1 — Supplementary file1 (PNG 201 kb) Gut metabolome profiles among ASD children with or without GI symptoms and TD children. [file 10803_2021_5066_MOESM1_ESM.png]
